# Promote identification or prevent expansion? The effect of uploader-viewer similarity on viewer inspiration in the context of short video community

**DOI:** 10.3389/fpsyg.2023.1120641

**Published:** 2023-03-31

**Authors:** Senhui Fu, Haiwen Dai

**Affiliations:** Department of Management, Guangdong University of Technology, Guangzhou, China

**Keywords:** short video community, short-video viewer inspiration, similarity effect, promote Identification, prevent expansion

## Abstract

**Introduction:**

Short video communities have proliferated recently, opening up a new channel for the promotion of online businesses. The main challenge in marketing with short videos is how to inspire consumers.

**Methods:**

This study employs viewer social identification and self-expansion as its entrance point to to explain how uploader-viewer similarity affects viewers’ short video inspiration. We use empirical investigation method based on the data of 310 short video viewers to test our conceptual model.

**Results:**

We found that there is a double-edged relationship between uploader-viewer similarity and short video inspiration. Particularly, uploader-viewer similarity enhances perceived social identification while reducing perceived self-expansion. The association between Uploader-viewer similarity and short video inspiration is moderated by viewers’ cognitive demands.

**Discussion:**

These results provide a new perspective for enterprises to manage the relationship between viewers and media.

## 1. Introduction

The “*Digital China Development Report (2020)*” published by the *National Internet Information Office of China* shows that the internet penetration rate has reached 70.4% by the end of 2020 and that the number of Chinese internet users has reached 989 million. According to data from the *China National Copyright Administration*, short video users made up 88.27% of all online users, and Chinese mobile internet users use short video applications more than a quarter of the time every day. Short videos appear to have replaced graphics and voice as the “third language” of the mobile internet and have been incorporated into people’s daily lives. For users, short videos are a relief valve for emotions, a vane for consumption, an assistant in life, and an aggregator of knowledge according to *2019 Short Video Marketing White Paper*, released by *Zhimeng Bejing Company*. For internet businesses, short videos are a new medium for promoting products and establishing and maintaining good relationships with their customers. The problem of how to successfully inspire customers to make purchases by capturing their interest while they watch videos still needs to be addressed. Researchers in the field of marketing have recently given the idea of consumer inspiration more consideration (Böttger et al., 2017).

In the context of short videos, individuals gain new ways of cognition, and they also fall into information cocoon (Peng and Liu, 2021) caused by individual limitations and system mechanisms. On the one hand, the individual’s attention resources are limited, unable to cope with massive amounts of information, and the cognitive subject has its unique mechanism; on the other hand, the popular similarity-based mechanism (such as the recommendation algorithm based on people or things recommendations based on similarity) may have an information cocoon effect. People enjoy the information ocean of short videos, the carnival of interpersonal communication, but ignore the discussion of cognitive dilemmas, or the weak reflection voice is submerged in the imagination of the technological utopia. In particular, the similarity elements and information acceptance mechanism are mined and woven under technologies such as smart recommendations and big data analysis. What is the influencing mechanism of viewers’ perception of similarity in short video context?

The similarity effect in social psychology (Byrne, 1971) helps us to solve this problem. Although the study of the similarity effect has achieved beneficial results, there are still three limitations. First, in the research context, a large number of studies have tested the similarity between buyers and sellers in the context of face-to-face interaction (Simons et al., 1970; Miller and Gerstein, 1984; Jiang et al., 2010). But few studies have focused on the similarity between uploaders and viewers in the short video context of the new business model. The uploader–viewer similarity will influence viewer inspiration. Therefore, it is necessary to explore whether this kind of interpersonal similarity effect still works in the new short video context. Second, from the perspective of research, the research on similarity and its consequences stays in a positive perspective (Guéguen et al., 2011; Brack and Benkenstein, 2012; Kachersky et al., 2014), ignoring the possible negative effects, which limits the comprehensive understanding of similarity effects to a certain extent. Third, the study lacks consideration of differences in individual cognitive motivations. Individual cognitive needs are an important factor influencing people’s decision-making (Cacioppo and Petty, 1982), which limits the application of similarity effects in the study of new media consumption behavior. From the point of view of entropy, cognition is the ability to reduce uncertainty by extracting new information, generating new information, improving knowledge and reflecting abilities to support the subject’s adaptability to the environment.

From the perspective of short video uploader–viewer similarity, this article studies the mechanism of similarity’s influence on viewer inspiration. From the two perception dimensions of increasing social identification and limiting self-expansion, we understand the role of similarity between short video uploaders and viewers and the moderating role of cognitive needs in it. The research focuses on the double-edged sword effect between uploader–viewer similarity and viewer short video inspiration; that is, uploader–viewer similarity has the positive effect of enhancing perceived social identification and at the same time has the negative effect of restricting the viewer’s self-expansion effect. At the same time, we explore the moderating role of viewers’ cognitive needs on the relationship between uploader–viewer similarity and viewer inspiration; specifically, the higher the cognitive needs, the more significant the negative impact of similarity on short video inspiration, and vice versa. In view of this, companies need to recognize the potential negative effects of uploader–viewer similarity in short video marketing to avoid the negative effect of similarity, apply the positive similarity effect to management practices, and promote user short video inspiration.

## 2. Theoretical background and hypotheses

### 2.1. The uploader–viewer relationship in short videos: From similarity attraction to similarity disgust

In sociology, the similarity effect describes a phenomenon in which people prefer people whose characteristics are similar to them in reality (Byrne, 1971; Zhao et al., 2022). People communicating with similar ones are smoother, understand each other better, and are more able to predict each other’s reactions more accurately. The similarity effect is the most powerful proof of relationships in social psychology (Berger, 1975). The positive effects of similarities in demographic characteristics (such as age, gender, income, and career), facial characteristics, and psychological characteristics (such as lifestyle and personality, attitudes, opinions, and preferences) have all been confirmed in the studies (Shen et al., 2010). Similarity can stimulate a sense of connection between oneself and others (Jiang et al., 2010), which is consistent with the principle of attraction in social media.

With the widespread use of the internet, researchers have begun to explore the positive role of similarity in the internet context. In the Facebook context, people are more willing to make friends with people who have accidental similarities (such as the same age, birthday, and hobbies) with them (Guéguen et al., 2011). Research on social media shows that the similarity between individuals is an important indicator of their future interactions (Crandall et al., 2008). In the context of short video, more and more videos are uploaded by common users. A user is said to be an uploader if it has uploaded at least one video (Yuan et al., 2011). Therefore, the uploader in this paper refers to the user, as we-media practice subject, who uploads video in the short video applications. The viewer in the short video community can communicate with the uploader or check reviews to obtain information about the uploader. Through long-time interaction, viewers can accurately identify whether they are similar to the uploader of the video.

We expect that in the context of the short video, uploader–viewer similarity will become an important variable in the realization of social behavior; specifically, the perception of similarity is conducive to activating viewers’ short video inspiration. The collection, recontextualization, and compression of short videos are not only information but also individual cognition. In the context of short videos, viewers are more likely to be influenced by similar people. Similarity can bring about mutual attraction, increase interaction, inspire a sense of connection, change attitudes, and promote the occurrence of implicit behaviors (Martin et al., 2013).

However, researchers such as Aron believe that in some situations, similarity does not have a relationship with attraction and may even destroy attraction (Aron et al., 1992). Evolutionary psychologists believe that humans identify with and prefer to mate with individuals of different genes in the course of evolution (Grob et al., 1998). From the perspective of individual self-development, the self-expansion model shows that people need to expand their self-efficacy to grow and develop themselves. Based on this assumption, individuals with differences can provide more resource information and are more attractive (Aron et al., 1992).

Therefore, as a general principle, similarity can indeed bring attraction, but in certain situations, differentiation is more attractive, and similarity at this time has a certain repulsive effect. Moreover, some aspects of similarity that can be attractive are also different from person to person. Because people are complicated in life, the psychology and behavior of viewers in the context of short videos are also different from those in real life. There is still a long way to go to study whether uploader–viewer similarity in short videos is attractive.

### 2.2. Viewer perception in short videos: From seeking social identification to pursuing self-expansion

Social identification is a person’s perception of the group to which he belongs, as well as the perception of oneself (Tajfel and Turner, 1979). The degree of individual identification with the group determines his perception of the group, which in turn affects his behavior. For example, individuals who highly identify with the group are more willing to cooperate closely with other members of the group. When people are aligned with the values of their group, they will focus on the interests and goals of the group and associate individual efforts and work roles with group values (Chadborn and Reysen, 2018). The influence of social identification on job performance can be explained by the individual’s cognition and emotional identification of the group, which can enhance the individual’s sense of self-efficacy.

On the short video platform, viewers gather under a loose community label to form a virtual community or circle. In the video platform, the gender, personality, age, status, education level, values, interests, and preferences of the video uploader are all elements that attract viewers and form social identification. Research on interpersonal attraction in social psychology has shown that people like others who share similarities with themselves because human beings are social animals. When people interact with similar others, they will experience smoother communication and better understanding, and they will be able to predict the reactions of the other person. In addition, similarity can stimulate a feeling of connection to others, which is consistent with the principle of attraction in social media. According to social response theory, which was proposed by Reeves and Nass (1996), people will apply the social norms in reality to computers that have human features. Therefore, online similarity can also stimulate connections and can lead to an increased willingness to interact. Viewers of short videos have formed self-awareness in the virtual community and can obtain clues by following a long period, thereby perceiving similarities with the video uploader (for example, common interests and preferences). Although the uploader and the viewer do not know each other face-to-face, long-term accompanying creates a social bond and experience social identification (Chadborn and Reysen, 2018).

However, it is worth noting that when viewers have a stronger willingness to participate, initiative, and a sense of efficacy, self-expanded cognition appears. Self-expansion refers to the psychological change or process of an individual through the expansion of self-identification into another person (Aron and Aron, 1986). In the context of interpersonal relationships, the main way people pursue self-expansion is to include others in themselves. After expanding the self, the individual sees others as part of the self and produces an illusion of overlapping self-other identities (Dys-Steenbergen, 2016). Over time, the resources, opinions, and identities of others are inspired by one’s self-concept. These principles are applied to the study of various relationship issues, including romantic love and group relationships. Self-expansion theory proposes two possible sources of self-expansion. One is to acquire new resources, perspectives, and identities, such as knowledge, social status, or relationships. The second source is joint participation in any novel, arousing activity, as long as they are not overwhelming or highly stressful (Aron et al., 1992).

In the context of short videos, when viewers interact with uploaders who provide new perspectives, resources, and identities in a short video context, they will find opportunities for self-expansion. Self-expansion theorists do not emphasize the motivation of future behavior but rather focus on the motivation of acquiring resources to expand personal self-awareness (Aron et al., 2013). Therefore, in the virtual community of the video platform, viewers obtain cultural capital through the platform and the video uploader’s ideas, concepts, or other resources and regard it as part of their self-worth. In this process, self-efficacy can be improved. When self-expansion occurs, the individual believes that he or she is more closely connected with others and that the quality of the relationship is higher (Cacioppo and Petty, 1982). These close social connections provide various resources, such as information sources and emotional support. Based on this, this article proposes the following hypotheses.

*Hypothesis 1*: Uploader–viewer similarity positively affects the viewer’s perception of social identification.

*Hypothesis 2*: Uploaded–viewer similarity negatively affects the viewer’s perception of self-expansion.

### 2.3. Short video inspiration under the cognitive dilemma of viewers

Viewer inspiration in the context of the short video refers to the inspiration that the short video brings to the viewer, which changes the viewer from “inspired by external factors (such as Aha, product A has so many usages!)” to “inspired to practice new ideas (such as purchase behavior occurs)” the activation of state transition (Böttger et al., 2017). Short video inspiration can prompt consumers to change from the idea of accepting marketing guidance to the inner pursuit of consumption-related goals.

Uploader–Viewer similarity not only increases perceived social identification (“the blade of benefit”) but also weakens perceived self-expansion (“the blade of disadvantage”). The positive side is that viewers can gain a sense of identity through interaction with similar uploaders. The process of interaction, feedback, exchange, and sharing of information between the uploaders and viewers of short videos create a sense of psychological security that is very important. Smooth communication increases positive emotions (Xiao et al., 2020), which in turn increases social identification (Huang et al., 2020). Uploaded-viewer similarity can relieve stress, relax the mood, resolve negative emotions, and trigger positive emotions. Previous studies have examined the influence of social identification on motivating individuals to think about their inner groups and found that a person’s identification power will affect his level of inspiration in a significant group. Second, social identification significantly increases the intensity, frequency, and perception components of speculative inspiration. As a result, uploader–viewer similarity will enhance social identification and further stimulate the generation of short video inspiration.

On the other hand, for the blade of disadvantage, uploader–viewer similarity can make viewers feel that their self-expansion is restricted. Although existing studies have pointed out that similarity can satisfy the individual’s sense of identification, belonging, and control from a cognitive perspective, this type of research is limited to situations where the individual’s sense of belonging and lack of control, such as a completely unfamiliar shopping environment, user experience of new products, etc. When the individual is at a normal level of control, the existence of uploader–viewer similarity will limit the individual’s perception and cannot expand itself. Improving self-efficacy by expanding one’s self is a basic motivation for people. Furthermore, the experiment of Aron et al. (2006) verified the hypothesis that “the possibility of developing relationships will weaken the effect of similarity on initial attractiveness.” The viewer’s inspiration in the context of short videos is a business model based on interpersonal relationships, which shows that viewers already exist or have the possibility of developing relationships in the future. When people expect to see someone again, they will be carefully considered when evaluating that person to avoid undesirable consequences rather than making quick decisions based on similarity. Strong group similarity hinders the free exchange of information and knowledge sharing between group members, and the sense of identification brought by good interpersonal relationships among group members helps promote the flow of knowledge and information. Therefore, uploader–viewer similarity limits the possibility of self-expansion, thereby reducing the viewer’s inspirational behavior.

Drawing on the self-extension model, in the short video platform community, the longer the relationship between the uploader and the viewer lasts, the less self-extension can be obtained, and vice versa, the more loyal they become (Cacioppo and Petty, 1982). If the short video uploader and the viewer are similar to each other and the viewer is familiar with the cognitive boundary of the uploader, the cognition will be limited due to too much similarity. If the video viewer and the uploader are very different, they cannot detect each other’s cognitive boundaries, new knowledge and information will continue to emerge, the information obtained from each other will be richer and multidimensional, and more information will be integrated to make decisions. It is also regarded as a more sensible choice. At this time, viewers are more likely to accept information from different video uploaders, thereby strengthening their self-expanding perception and then generating positive short video-inspirational behaviors.

The current literature on similarity effects focuses on the positive effects of similarity, such as enhancing attractiveness, enhancing persuasiveness and compliance, enhancing trust and preference, and enhancing social relations (Byrne, 1971; Jiang et al., 2010; Fu et al., 2018), there is little research mentioning whether uploader–viewer similarity may have negative effects. From the hypothesis that similarity brings compliant behavior (Byrne, 1971), uploader–viewer similarity may bring a sense of restraint and restriction to viewers, which in turn leads to negative attitudes. In addition, some studies have found that in certain situations, differences are more attractive (Hean et al., 2006). The uploader–viewer similarity not only has a unilateral positive impact on the viewer’s short video inspiration but may also have a negative impact; that is, it has a “double-edged sword” path with both gain and loss. The presentation or recommendation of information in the short video community will exacerbate this “double-edged sword” effect. Synthesizing the “double-edged sword” path, this article proposes the following hypotheses.

*Hypothesis 3a*: Perceived social identification positively affects viewers’ short video conceptual inspiration.

*Hypothesis 3b*: Perceived social identification positively affects viewers’ short video action inspiration.

*Hypothesis 4a*: Perceived self-expansion positively affects viewers’ short video conceptual inspiration.

*Hypothesis 4b*: Perceived self-expansion positively affects viewers’ short video action inspiration.

### 2.4. The moderating role of the viewer’ cognitive need

The viewer’s cognitive need will moderate the relationship between uploader–viewer similarity and the viewer’s self-expanded cognition. The need for cognition (NFC) is an important personality trait that reflects differences in individual cognitive motivation. Cognitive needs are the tendency of an individual to participate and enjoy thinking. It mainly considers whether the individual is willing to think actively when facing cognitive tasks (Cacioppo and Petty, 1982). High NFC tends to process complex information, pay attention to the collection and processing of information, and discover the logic between phenomena. Relatively speaking, people with low cognitive needs tend to think quickly, have shallow information processing, and prefer explicit information. The cognitive needs of viewers are significantly positively correlated with the behavior of using Twitter to obtain information (Hughes et al., 2012). Moreover, individuals with high NFC are more able to integrate messages including negative frames, effectively integrate information, and expand themselves, which is conducive to short video inspiration for viewers. Based on this, it can be inferred:

*Hypothesis 5a*: The higher the viewer’s cognitive needs are, the stronger the negative impact of uploader–viewer similarity on the viewer’s self-expansion.

*Hypothesis 5b*: The lower the viewer’s cognitive needs are, the less significant the influence of uploader–viewer similarity on viewer self-expansion.

The Stimuli-Organism-Response Model (SOR) provides a framework that describes “uploader–viewer similarity” as a stimulus, while “perceived social identification” and “perceived self-expansion” are different types of organism, and the “viewer’s inspiration” is a type of response.

For the stimuli, previous research on short video shopping has indicated that the experience of users in differs from offline shopping, as consumers have social interactions with sellers (Wang, 2021) and other users online (Meng and Leung, 2021). Once a user interacts with sellers and other users online, it is likely that they can know more information about the product or brand, further more likely to get inspiration to buy. Thus, similarity between uploader and viewers in the sort video context is an important stimulus that influences viewers’ intentions and behaviors.

For the organism, most human behaviors are driven by mental states, which are often affected by how we relate to a specific stimulus. This type of organism includes cognitive and affective reactions. Previous environmental psychology research defined cognitive reactions as ‘the mental process occurring in individuals’ minds when they interact with the stimulus’(Eroglu et al., 2001), and affective reactions are related to individuals’ emotional responses when they are stimulated by the environment (Sun and Zhang, 2006). In this context, we introduce perceived self-expansion and perceived social identification to explain viewers’ cognitive and affective reactions when interacting with the uploader in the short video context.

Consistent with the S-O-R model, the response represent the final outcomes and decisions of users based on cognitive and affective reactions and include approach or avoidance behaviors (Sherman et al., 1997). In the context of short video, the response has two important aspects, namely, inspired-by and inspired-to Böttger et al. (2017). Thus, this paper explores the effects of uploader–viewer similarity on short video from the perspectives of viewer’s perception.

## 3. Research design

### 3.1. Data collection

“Short video” is a relatively new social application based on mobile intelligent terminals. In this study, convenience sampling was conducted among active users of short videos, questionnaires were compiled through the *Questionnaire Star*^1^, and we choose users in *Douyin, Kuaishou, and Xiaohongshu*, the typical social commerce applications for reviewers to watch short videos and do some purchasing in Mainland China, as the participants. The short videos are social networking-based communities, where viewers can directly communicate with the uploader or other viewers in the same video community. The viewers’ idea of purchasing decisions are often influenced by the uploader’s presentation and recommendation. Meanwhile, these short video applications are also e-commerce, thus, there is a direct path or a link for purchasing online for all viewers. Therefore, it is common knowledge among viewers that these applications are online communities where people can easily communicate with the uploader, also can purchase the product in these communities.

Because this study was conducted in Mainland China, the scales in the questionnaire went through a translation and back translation process with the assistance of two doctoral students. First, one student translated the instruments from English to Chinese, and then, the other student translated them from Chinese to English. The two English versions of the scales were compared, and all inconsistencies were resolved to improve the quality of the questionnaire. Further, we invited experts, including professors and doctoral students who majored in psychology and marketing, to review the questionnaire to examine the face validity of the survey instruments, refine the questionnaire wordings, assess logical consistencies, judge the ease of understanding, and identify areas for improvement. Overall, the questionnaire was viewed as concise and easy to complete. They also proposed several suggestions for the formatting and wording of the questions, which were incorporated in the revised version of the questionnaire.

This study was introduced as an “opinion survey,” and the respondents were asked to recall their recent short video shopping experiences influenced by the uploaders that they frequently focus on these applications. Then, they were asked to complete the questionnaire based on their own experiences or perceptions. An opinion survey is commonly used to understand how respondents feel about target objects (e.g., Bagozzi and Dholakia, 2006; Shen et al., 2014), i.e., short video shopping activities for this study. As such, a screening question asked if potential respondents had watched video and purchased products in these applications, such as *Douyin, Kuaishou, and Xiaohongshu*, to ensure that all successful respondents had prior experience with short video shopping activities. This method is consistent with previous studies that adopted screening questions to identify the most appropriate respondents (e.g., Cheung and Lee, 2009).

Data were collected from July 10, 2021, to August 10, 2021, and a total of 316 questionnaires were collected. Among them, six questionnaires were deleted by filling in “Do you fill in seriously?” with “No” on the last question, so the number of valid questionnaires was 310. As the survey subjects were active viewers, the number of questionnaires could not be controlled. 310 is the maximum number that can be collected within a limited time. Other methods can be adopted to collect more questionnaires in the future.

Note that a possible concern for our study is non-response bias, which occurs when there is bias from significant differences between non-respondents and respondents. Because it was not possible to compare these two groups of users in this study, we followed prior research (e.g., Al-Qirim, 2007) and compared the demographics for the early (the first 50) and late (the last 50) respondents. This approach views late respondents as representative of non-respondents (Karahanna et al., 1999). The results showed that there were no significant differences. Hence, non-response bias is not a critical concern in this study.

According to the Pew Duggan (2015) *Short Video Usage Report*, the typical viewer image of short videos is 18 to 29 years old, showing the characteristics of high education, high income, urban residents, etc. *The 2014 Chinese Social Application Viewer Behavior Research Report* released by the China Internet Network Information Center also confirmed that short video viewers are “younger and highly educated.” Nearly 80% of the respondents in this study were under 30 years old (*N* = 304, SD = 1.20), and 78% of the respondents had a bachelor’s degree or above (*N* = 304, SD = 1.06). The proportions of male and female viewers were 47.8 and 52.2%, respectively. Therefore, the sample characteristics of this study can reflect the overall characteristics of short video viewers.

### 3.2. Definition and measurement of variables

We use the following Table 1 to illustrate the definition and measurement of the variables, including the viewer’s short video inspiration (inspired-by and inspired-to), uploader–viewer similarity, perceived social identification, perceived self-expansion, and the viewer’s need for cognition.

**Table 1:** Definition and measurement of variables.

Variable	Definition	Measurement
Inspired-by	Being inspired by external factors (Böttger et al., 2017).	1. My imagination was stimulated
		2. I was intrigued by a new idea
		3. I unexpectedly and spontaneously got new ideas
		4. My horizon was broadened
		5. I discovered something new
Inspired-to	Being inspired to practice new ideas (Böttger et al., 2017).	1. I was inspired to buy something
		2. I felt a desire to buy something
		3. My interest to buy something was increased
		4. I was motivated to buy something
		5. I felt an urge to buy something
Uploader–viewer similarity	The similarities between short video uploaders and viewers (Fu et al., 2018).	1. When I watch the short video, I often interact with the uploaders who are at the same age as me
		2. When I watch the short video, I interact with the uploaders who regularly are in the same class as me
		3. When I watch the short video, I often interact with the uploaders who frequently have a similar education level to me.
Perceived social identification	Viewers regard themselves as a member of a certain interpersonal circle of short videos (Tse et al., 2012).	1. I pay attention to and actively participate in activities when I watch the short video
		2. I am willing to make efforts to maintain my image when I watch the short video
		3. I like the uploaders who interact with me in short videos.
Perceived self-expansion	The psychological change or process that the viewer inspires another person through the expansion of self (Gorlier and Michel, 2020).	1. Short videos help me expand my horizons.
		2. I think short videos teach me many new things.
		3. This short video band gives me many new experiences.
The viewer’s need for cognition	The viewer’s tendency to participate in and enjoy thinking (Cacioppo and Petty, 1982).	18 items such as “I prefer complex questions to simple ones,” “I like to deal with problems that require much thinking,” and the reverse scoring items such as “I do not want to think until I have to.”

The Cronbach’s alpha value of each variable was above 0.80, indicating high reliability. All items were measured using a 7-point Likert scale ranging from “1 = strongly disagree” to “7 = strongly agree.” Online surveys conducted without random assignment may increase the likelihood of individual differences in the system, affecting the results. Therefore, this study includes some general control variables to measure the characteristics of viewers on social networking sites, such as gender, income, and residence. Experience with internet use was also taken into consideration.

### 3.3. Data analysis and results

In this study, the least square method (PLS) was used to calculate the measurement model and structural model, and SPSS 26.0 software and its supporting plug-in PROCESS were used to analyze the data for descriptive statistical analysis and regression analysis.

Descriptive statistics showed that 12.62% of the samples using short videos (N = 310) were mainly used to acquire knowledge information; 24.35% for social communication; 52.43% for leisure and entertainment; and 10.60% for shopping. In terms of the use frequency of short videos, 5.81% of users were less than 1 h per day; 36.13% were1-3 h per day; 45.81% were 3–6 h per day; 12.26% more than 6 h a day; and 0 people choose “never.” In terms of internet using experience, 3.87% were less than 1 year; 7.74% were 1–3 years; 12.26% were 3–5 years; 27.1% were 5–8 years; and 49.03% were over 8 years.

#### 3.3.1. Measurement model

The scale reliability was tested by Cronbach’s α and complex reliability (CR). The results showed that Cronbach’s α coefficients of all variables were greater than 0.8 and the CR was greater than 0.9, indicating that the scale had good internal consistency and a high-reliability level. The calculation of the standard load of the measurement item showed that the load of all items was greater than 0.7, and the average variance extraction value (AVE) of the latent variable was greater than 0.5, indicating that the variable measurement item had good convergence validity (see Table 2).

**Table 2:** Results of the confirmatory factor analysis.

Variable	Items	Cronbach’s α	Composite reliability (CR)	AVE
Uploader–viewer similarity	3	0.905	0.928	0.826
Perceived social identification	3	0.875	0.921	0.796
Perceived self-expansion	3	0.836	0.917	0.783
Inspired-by	5	0.920	0.945	0.841
Inspired-to	5	0.915	0.936	0.837
Need for cognition	18	0.852	0.903	0.674

The square root of the latent variable AVE on the diagonal of the table matrix is greater than the correlation coefficient between variables (see Table 3), indicating that the scale has good discriminative validity.

**Table 3:** Means, standard deviations, and correlations.

Variable	Mean	S.D.	1	2	3	4	5	6
1. S	3.395	1.135	**0.909**					
2. SI	4.225	1.231	0.125	**0.892**				
3. PSE	4.346	1.143	−0.467	−0.131	**0.885**			
4. IB	3.798	1.215	0.334	0.385	0.445	**0.917**		
5. IT	3.623	1.217	0.420	0.422	0.486	0.570	**0.915**	
6. NFC	3.459	1.245	0.455	0.513	0.395	0.421	0.336	**0.821**

Diagonal values in bold are the square root of the AVEs.

#### 3.3.2. Structural model

Partial least squares structural equation modeling software SmartPLS 3.0 and SPSS 26.0 were used for testing. In the structural model, the explanatory validity coefficient *R*^2^ of perceived social identification was 0.601, the explanatory validity coefficient *R*^2^ of perceived self-expansion was 0.324, the explanatory validity coefficient *R*^2^ of short video conceptual inspiration was 0.456, and the explanatory validity coefficient *R*^2^ of short video action inspiration was 0.472, indicating that the structural model had the good explanatory ability. For the calculation of endogenous variable prediction correlation *Q* (Aron et al., 1992), 0 < *Q*^2^ < 0.15 indicates a weak correlation, 0.15 ≤ *Q*^2^ < 0.35 indicates a good correlation, and *Q*^2^ ≥ 0.35 indicates strong correlation. By calculation, the predictive correlation *Q*^2^ = 0.568 for perceived social identification, 0.336 for perceived self-expansion, 0.345 for short video conceptual inspiration, and 0.387 for short video action inspiration have good correlation strength. The goodness of fit (GoF) was used to identify the goodness of fit of the partial least squares structural model. 0.1 < GoF < 0.25 indicated low goodness of fit, 0.25 ≤ GoF < 0.36 indicated good goodness of fit, and GoF ≥ 0.36 indicated high goodness of fit. According to the calculation, the GoF of perceived social identification, GoF of perceived self-expansion, GoF of short video conceptual inspiration, and GoF of short video action inspiration in this study are 0.575, 0.345, 0.423, and 0.446, respectively, indicating that the model has good goodness of fit. See Table 4 for details.

**Table 4:** Structural model evaluation.

Variable	Explanatory power (*R*^2^)	Predictive correlation (*Q*^2^)	Goodness of fits (GoF)
Social identification	0.601	0.568	0.575
Self-expansion	0.324	0.336	0.345
Inspired-by	0.456	0.345	0.423
Inspired-to	0.472	0.387	0.446

#### 3.3.3. Hypotheses testing

**Figure 1:**
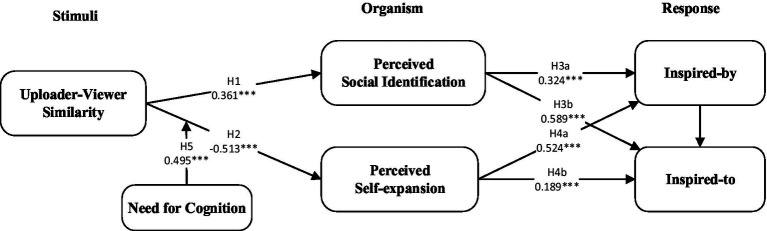
The research framework (***p < 0.001).

The partial least squares algorithm of structural equation modeling software SmartPLS 3.0 and bootstrapping (5,000 times) were used to analyze the path relations and indirect effects assumed in this study. Figure 1 and Table 3 show the results of data testing. Short video uploader–viewer similarity has a significant positive effect on perceived social identification (*β* = 0.361, *p* < 0.001). Short video uploader–viewer similarity had a significant negative effect on perceived self-expansion (*β* = −0.513, *p* < 0.001), and H1 and H2 were verified. Perceived social identification positively affected both conceptual inspiration and action inspiration (*β* = 0.324, *p* < 0.001; *β* = 0.589, *p* < 0.001), and H3a and H3b were validated. Both conceptual inspiration and action inspiration were positively affected by perceptual self-expansion (*β* = 0.524, *p* < 0.001; *β* = 0.189, *p* < 0.001), and H4a and H4b were verified.

In this study, the bootstrapping method was used to test the mediating model of viewers’ social identification and self-expansion. A total of 5,000 samples were repeatedly selected with a 95% confidence interval. Model 4 was used to test the mediating model of “perceived social identification,” and Model 7 was used to test the moderated mediating model of “perceived self-expansion.” The indirect effect of perceived social identification on short video conceptual inspiration was 0. 362, *p* < 0.001, 95% confidence interval did not include 0. The indirect effect of perceived social identification on short video action inspiration was 0.478, *p* < 0.001, 95% confidence interval did not include 0. The indirect effect of similarity on short video conceptual inspiration was −0.413, *p* < 0.001, 95% confidence interval excluding 0. The indirect effect of perceived self-expansion on short video action inspiration was −0.205, *p* < 0.001, 95% confidence interval excluding 0. Table 5 shows that perceived social identification and perceived self-expansion have significant mediating effects.

**Table 5:** Path analysis.

Indirect path	Indirect effect	SE	*t*-value	95% CI
Similarity→Social identification→Inspired-to	0.362***	0.039	5.418	[0.141, 0.292]
Similarity→Social identification→Inspired-to	0.478***	0.043	7.214	[0.235, 0.427]
Similarity→Self-expansion→Inspired-by	−0. 413***	0.027	4.103	[−0.175, −0.041]
Similarity→Self-expansion→Inspired-to	-0. 205***	0.019	3.98	[−0.165, –0.050]

***p < 0.001.

#### 3.3.4. The moderating effect analysis

Table 6 shows that the uploader–viewer similarity regression coefficient was −0.312, *p* < 0.001. The need for cognition regression coefficients was 0.236, *p* <0.001, playing a significant role in the short video context. The variable of need for cognition plays a regulatory role in the relationship of the uploader–viewer similarity and self-expansion when the viewer cognitive demand is higher; While the uploader–viewer similarity has a stronger negative effect on viewers’ self-expansion; When the viewer’s cognitive needs were lower, the similarity between short video uploader and viewer had no significant effect on viewers’ perceived self-extended, and H5a and H5b were verified.

**Table 6:** The moderating effect analysis.

Dependent variable	Independent variable	*β*	*t*-value	Coefficient of significance
Perceived	Uploader–viewer similarity	−0.312	−10.96***	0.000
Self-expansion	Need for cognition	0.236	8.71***	0.000

Adjusted *R*^2^ = 0.574, *F* = 52.786, *p* = 0.000. ***p < 0.001.

## 4. Conclusion and discussion

### 4.1. Discussion of key findings

From the perspective of uploader–viewer similarity, this paper explores the mechanism of viewer inspiration in the short video community and explains the cognitive dilemma in short video usage. Through the questionnaire survey and analysis of short video viewers, it is found that uploader–viewer similarity has a positive effect on viewers’ social identification but has a negative effect on viewers’ self-expansion. Both social identification and self-expansion have a positive effect on short video inspiration. In other words, the similarity between short video uploaders and viewers can improve the social identification of viewers but limit their self-expanding cognition. Therefore, the positive role of uploader–viewer similarity should be played in practice, and its negative role should be avoided.

First, the interpersonal similarity is an important source of attraction for viewers’ inspiration for short videos. The results show that uploader–viewer similarity can explain 45.5% of the variation in viewers’ conceptual inspiration and 47.2% of the variation in viewers’ action inspiration in short videos, indicating that uploader–viewer similarity plays an important role in short videos. Second, uploader–viewer similarity has a “double-edged sword effect,” which has both positive and negative effects. Third, cognitive needs regulate the relationship between uploader–viewer similarity and self-extended cognition.

The internal logic of this mechanism, on the one hand, is that the framework of the short video fits the logic of contemporary communication. Similarity enhances three characteristics of short video inspiration: the need for communication (i.e., the motivation to maximize communication satisfaction and interaction), assumed similarity (the perception that uploaders and viewers using the same medium are close to each other), and simplified social cues (nonverbal and paralingual cues lacking communication). As Van Dijk points out in his book “Connected: A Critical History of Social Media” when discussing the relationship between connectedness and connectivity of social media platforms, the convergence of interpersonal connection and automatic connection occur when people’s social activities are translated into algorithmic recommendations of the platform.

According to media compensation theory, communication can be achieved naturally through short videos. Due to the same or similar internal schema of communication, although media changes the external characteristics of communication (transregional and transtemporal), it does not change the essence of communication. The similarity between short video uploaders and viewers makes communication smoother. Although there is a certain amount of information loss in this process compared to face-to-face communication, such simplified social cues can also achieve relationship connection in a shorter cognitive time. For example, similarity factors are more likely to trigger altruistic consciousness and promote the occurrence of online prosocial behavior. This has implications for reshaping the symbiotic relationship between individuals and social communities as well as a way to deal with risks.

On the other hand, uploader–viewer similarity benefits social capital. For those who find it difficult to find social capital offline, the internet can be used as a mechanism or means of compensation (Baym, 2010). In particular, virtual community users get together due to common interests, hobbies and values, resulting in the “proximity effect,” thus stronger social identification and cohesion and more spiritual comfort and psychological support. Therefore, under the action of the communication logic of short videos and the internal and external factors of social capital acquisition, the similarity between uploader and viewer becomes the motivation for inspiration of short videos.

### 4.2. Theoretical contributions

First, the “similar-attraction theory” is a well-known theory of interpersonal attraction. Similarity leads to attraction, promotes interaction, and provokes feelings of connection. This study also verified that the “similarity effect” in social psychology can activate viewers’ short video inspiration in a short video context.

Second, existing studies focus more on similarity in face-to-face situations and focus on the biased positive conclusion that “similarity leads to attraction,” ignoring the negative effects of similarity. In particular, it does not explain why in some situations similarity plays a less important role in attractiveness or even prefers dissimilar people. Based on the contradiction between viewers’ social identification and self-expansion, this study suggests that similarity can both improve viewers’ social identification and weaken their self-expansion. In other words, similarity brings social identification to viewers, while dissimilarity brings self-expansion. Therefore, from the perspective of the self-expansion dimension, uploader–viewer similarity will make viewers experience a sense of alienation in online communication, resulting in the phenomenon of network exclusion.

Third, according to learning theory, learning is a kind of dialog that includes the internal dialog of individuals and the external dialog of interpersonal individuals. Hrastinski further believes that internet learning is also a process of participating in interaction (Hrastinski, 2009). Some scholars (Carver and Scheier, 1987) put forward two kinds of self-awareness, including public self-awareness and private self-awareness. Individuals with high self-consciousness pay attention to others’ evaluations of themselves and tend to carry out strict impression management; High-ego individuals focus on their internal standards, experiences, and perspectives. In short videos, ego consciousness with high cognitive needs comes into play, and it has low requirements for similarity and ignores impression management in virtual communities, resulting in deviant behaviors, such as seeking cognitive resources outside the circle.

### 4.3. Practical implications

First, the short video communities and platforms should give full play to the positive role of uploader–viewer similarity, present the similarity between viewers, establish a sense of connection between viewers, increase social identification and activate the inspiration of short videos.

Second, there are many factors affecting network exclusion, among which information acquisition is an important one. When viewers obtain less and less different information in the video, it means that others are no longer important to their self-expansion.

Third, short video enterprises should aware of viewers with high cognitive needs will choose more diverse and higher-quality dialogs or interactions to improve their self-expansion ability. Therefore, for viewers with high cognitive needs, the suggestion or presentation of uploader–viewer similarity should be reduced; for viewers with low cognitive needs, the use of uploader–viewer similarity can be increased. Especially with the deepening of the interaction between viewers and media, it is necessary to explore various specific cognitive elements of viewers in the context of short videos in a more diversified way, which provides a new perspective for further research on the relationship between viewers and media.

## 5. Limitations and future directions

The shortcoming of this study is that cross-sectional data are adopted. Future investigations can be conducted at multiple time points in the same short video community, and the causal relationship between similarity and short video inspiration and other variables can be explored through statistical analysis of data at different stages. In addition, future research can also try to use more subdivided short video websites, explore the cognitive differences of viewers in different cultural contexts, and explore the influence of the similarity effect between uploaders and viewers on viewers’ cognition and relationship.

## Data availability statement

The raw data supporting the conclusions of this article will be made available by the authors, without undue reservation.

## Author contributions

SF made substantial contributions to the conception of the work, the experimental design, the acquisition of data, the analysis and interpretation of data, and drafting the manuscript and agreed to be accountable for all aspects of the work in ensuring that questions related to the accuracy or integrity of any part of the work are appropriately investigated and resolved. HD made substantial contributions to the analysis and interpretation of data and revising the manuscript critically. All authors contributed to the article and approved the submitted version.

## Funding

This research was supported by Philosophy and Social Science Planning Project of Guangdong Province, China (GD20YGL02) and Natural Science Foundation of Guangdong Province, China (2021A1515011438).

## Conflict of interest

The authors declare that the research was conducted in the absence of any commercial or financial relationships that could be construed as a potential conflict of interest.

## Publisher’s note

All claims expressed in this article are solely those of the authors and do not necessarily represent those of their affiliated organizations, or those of the publisher, the editors and the reviewers. Any product that may be evaluated in this article, or claim that may be made by its manufacturer, is not guaranteed or endorsed by the publisher.

## Footnotes

^1^ www.sojump.com
